# Detection Ewingella americana from a patient with Andersson lesion in ankylosing spondylitis by metagenomic next-generation sequencing test: a case report

**DOI:** 10.1186/s12891-024-07680-y

**Published:** 2024-07-20

**Authors:** Hui Wu, Xiaoyun Wu, Tianlong Wu, Xinxin Miao, Sikuan Zheng, Guanfeng Huang, Xigao Cheng

**Affiliations:** Department of Orthopedic Surgery, The Second Affiliated Hospital, Jiangxi Medical College, Nanchang University, Nanchang, Jiangxi 330006 China

**Keywords:** Andersson lesion, Ankylosing spondylitis, Case report, Ewinia americana, mNGS

## Abstract

**Background:**

Andersen’s lesion (AL) is a rare complication of ankylosing spondylitis (AS), characterized by nonneoplastic bone destruction, typically manifested as bone destruction and sclerosis in the vertebral body and/or intervertebral disc area. At present, there is no consensus on the pathology and etiology of AL. Repeated trauma, inflammation in essence and part of the natural history of Ankylosing spondylitis itself are the most widely recognized theories of the etiology of AL. However, positive bacteria cultured in bone biopsy of Andersen’s lesion (AL) in Ankylosing spondylitis patients are extremely rare. Herein, we report a rare case of detecting Ewingella americana from a patient with Andersson lesion in ankylosing spondylitis by Metagenomic Next-Generation Sequencing (mNGS) Test.

**Case presentation:**

This case involved a 39-year-old male with a history of AS for 11 years, who developed AL (T11/12) in the thoracic vertebrae. After sufficient preoperative preparation, we successfully performed one-stage posterior approach corrective surgery and collected bone biopsies samples for examination. Cultured bacteria were not found, and pathological histology indicated infiltration of inflammatory cells. However, it is worth noting that we discovered a gram-negative bacterium, the Ewingella americana, through mNGS testing. Further histopathological examination suggests chronic inflammatory cell infiltration. After one-stage posterior approach corrective surgery, the patient’s condition significantly improved. At the 6-month follow-up, the pain significantly decreased, and the patient returned to normal life.

**Conclusion:**

We detected Ewinia americana in the bone biopsies of Andersson lesion (AL) in ankylosing spondylitis patient by mNGS.

## Introduction

Ankylosing spondylitis mainly affects the sacroiliac joint and spine, with clinical manifestations characterized by pain, lack of segmental movement, and spinal stiffness, and finally ends up with osteopenia and vulnerability to trauma [1, 2]. It was first reported by Anderson in 1937 that as AS lesions develop over the long term, non-invasive vertebral and intervertebral disc destructive lesions may occur, which are defined as Anderson lesions (AL) [3]. Since then, a series of terms have been used to describe the destructive lesions of ankylosing spondylitis. For example, spondylodiscitides, destructive spinal disease, spinal pseudoarthrosis, and Andersen’s disease. This phenomenon is mainly due to the lack of consistency between the etiology and pathology of the lesion. However, the inflammatory nature and partial natural history of ankylosing spondylitis itself and traumatic lesions are currently recognized as the two main theories [4–6]. Although the imaging findings are extremely similar to pyogenic or tuberculous infection, there is not enough evidence to show that AL has pyogenic or tuberculosis infection [4]. To our knowledge, there are few literature reports on the cultivation of bacteria in the bone biopsy of Andersen’s disease (AL) in patients with ankylosing spondylitis. Herein, we report a rare case of detecting Ewingella americana from a patient with Andersson lesion in ankylosing spondylitis by mNGS.

## Case report

**Fig. 1:**
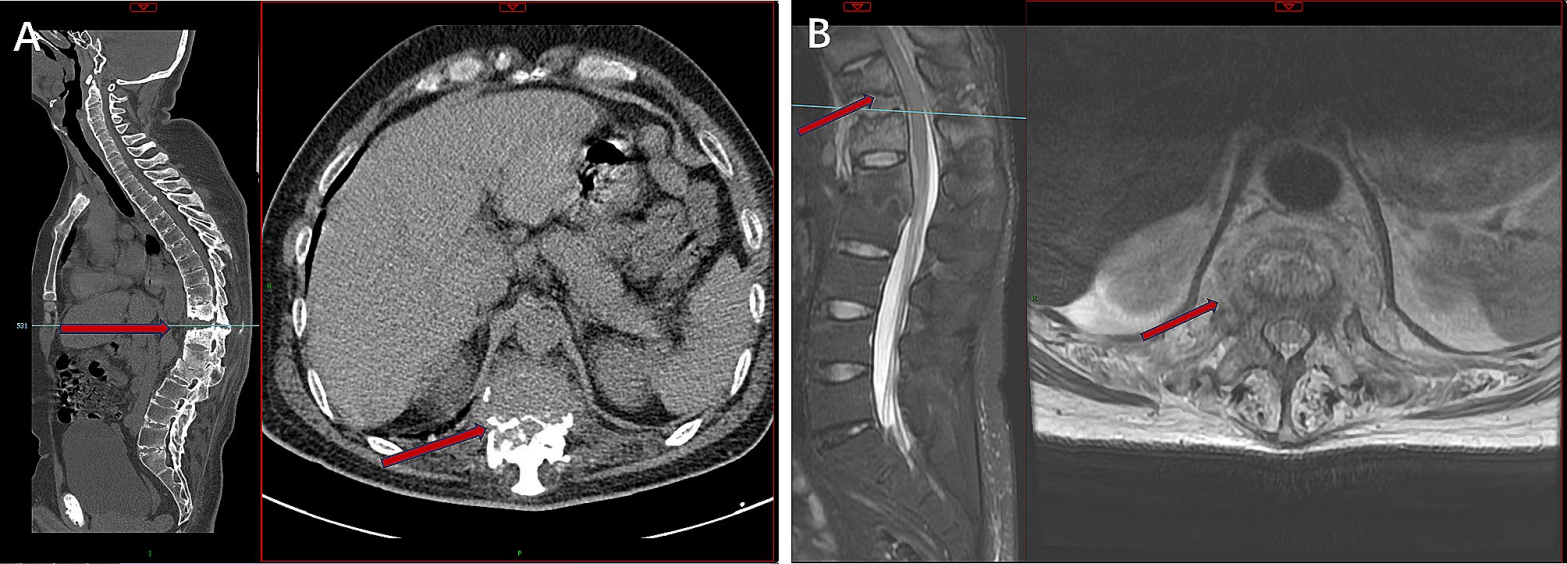
**A**: The sagittal and axial computed tomography scans indicate significant thoracic kyphosis and osteolysis in the T11/12 intervertebral space (arrow), with irregular sclerotic zone surrounding it and lesions extending to the posterior elements. **B**: Magnetic resonance imaging shows erosion of the intervertebral disc and adjacent endplates between T11 and T12 (arrow)

**Fig. 2:**
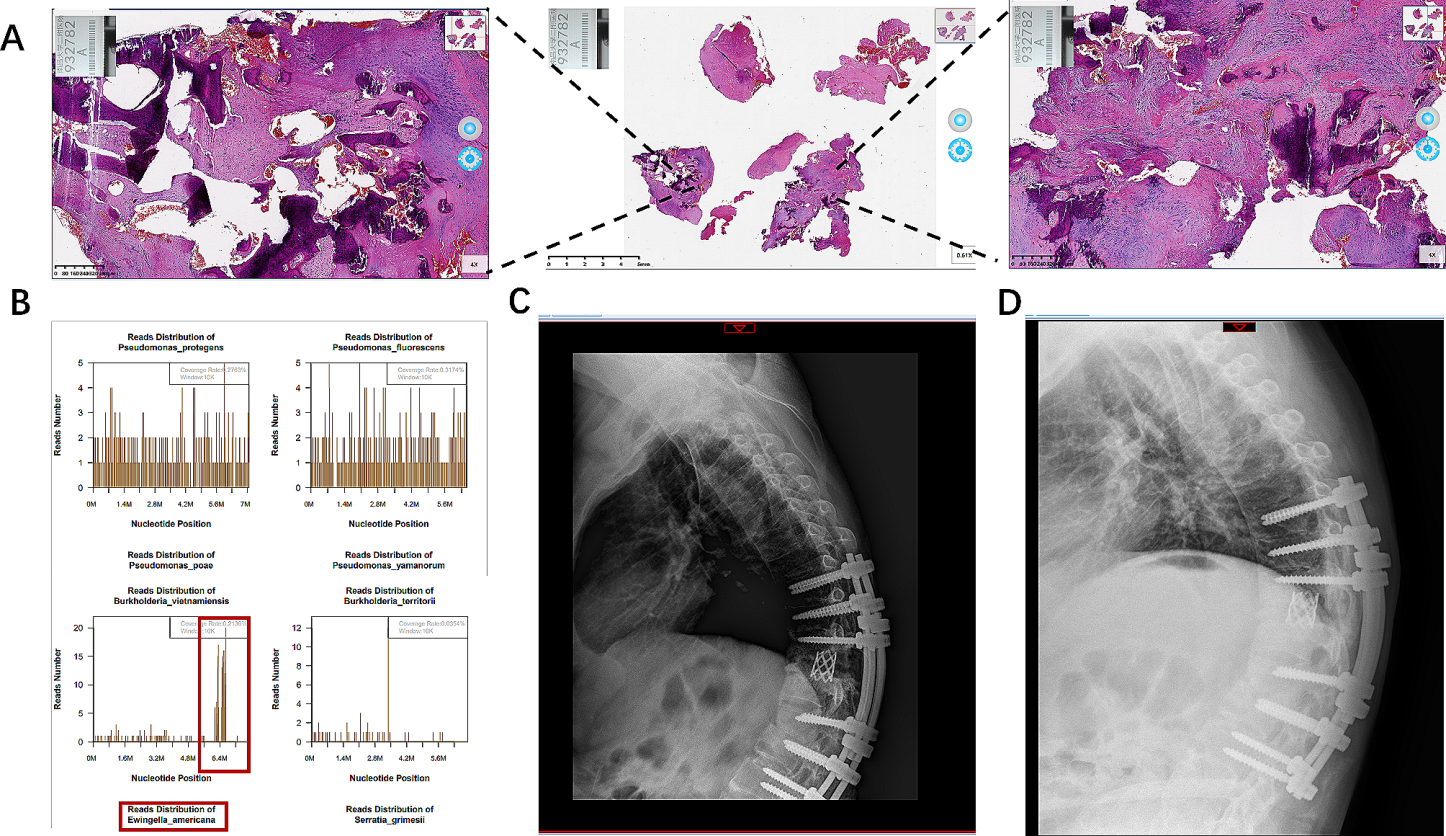
**A**: Microscopic histopathological examination revealed fibrocartilage and bone tissue, fibrous necrosis, granulation tissue formation, and chronic inflammatory cell infiltration. **B**: Through Metagenomic Next-Generation Sequencing Test, Ewingella americana was identified in specimens collected from bone biopsies. **C**: Spinal lateral radiography at the 2-weeks post-surgery. **D**: Spinal lateral radiography at the 3-months follow-up

A 39-year-old male was diagnosed with a history of AS 11 years ago and was admitted to the hospital about 2 months ago due to severe back pain and inability to walk on his own. He received conservative treatment, including exercise, non-steroidal anti-inflammatory drugs (NSAIDs) and long-term use of steroid hormones, but his irregular medication resulted in poor efficacy. At the time of admission, the patient had clear consciousness, good mental condition, physical examination indicated that their lower limb muscle strength reached level 4+, respectively. His body temperature was normal and noted to be 36.9°. Peripheral blood tests indicated that an increase in C-reactive protein (CRP) and erythrocyte sedimentation rate (ESR) levels to 13 mg/dL (normal range < 0.05 mg/dL) and 99 mm/h (normal range < 20 mm/h), respectively. However, the white blood cell (WBC) count was normal. No bacteria discovered in blood culture. The sagittal and axial computed tomography scans indicate significant thoracic kyphosis and osteolysis in the T11/12 intervertebral space, with irregular sclerotic zone surrounding it and lesions extending to the posterior elements (Fig. 1A). Magnetic resonance imaging shows erosion of the intervertebral disc and adjacent endplates between T11 and T12 (Fig. 1B). However, inquire about the patient’s medical history, and the patient and their family deny any history of recent trauma or previous pyogenic spondylitis. After sufficient preoperative preparation, we plan to undergo one-stage posterior approach corrective surgery and collected bone tissue samples for examination. From T8 to L4 levels, midline incision is performed through a posterior approach, followed by laminectomy at T10 and T12 levels and vertebral body resection at T11 level to alleviate spinal cord compression. The gap between the ventral defects is filled with a mesh cage to provide ventral support. Finally, by applying pressure to the vertebral body, the mesh cage is fixed in the appropriate position. After massive irrigation, to prevent instability, pedicular screw fixation was performed at the T8, T9, T10, T12, L1 and L2 vertebrae. The deformity of spinal kyphosis has been well corrected. During the surgery, we observed a large amount of sclerotic and discontinuous bone tissue. Bone tissue was collected and confirmed through microbiology, histology, and mNGS. Irrigation with a large amount of physiological saline solution containing cefoperazone sodium and sulbactam sodium. The surgery was very successful without any complications related to the surgery. Conventional bacterial culture did not detect any bacteria. Microscopic histopathological examination revealed fibrocartilage and bone tissue, fibrous necrosis, granulation tissue formation, and chronic inflammatory cell infiltration (Fig. 2A). However, through mNGS, Ewingella americana was identified in specimens collected from bone biopsies (Fig. 2B). At the 2-weeks and 3-months follow-up, radiologic findings showed that the screws were well maintained (Fig. 2C-D). In addition, the ESR and CRP levels had markedly dropped to 37 mm/h and 0.6 mg/dL, respectively. The back pain improved progressively after surgery. At the 6-months follow-up, the symptoms gradually improved, the pain significantly decreased, and the patient returned to normal life.

## Discussion

The etiology of AL is still unclear, but the debate about its etiology has never stopped [4]. Currently, two main theories have been widely recognized. Firstly, they are primary inflammations and are part of the natural history of ankylosing spondylitis. The second type is that they are traumatic lesions and form pseudo joints [7, 8]. Research has shown that biopsies of AL in the late stage of the disease show reactive new bones, as well as cartilage nodules or pseudoarthrosis around micro-fracture, suggesting that repeated mechanical stress may be the initial and potential factor for the development of AL; However, repeated pressure from minimally invasive injuries cannot well explain early AL or the development of AL in children [7, 9]. As is well known, inflammatory markers in AL patients are usually elevated, and imaging findings are highly similar to purulent spondylitis or tuberculous osteomyelitis. Therefore, some scholars have proposed that infection is the main etiology, but almost no scholars have cultured positive pathogens from bone tissue [4]. In addition, the histopathological results of AL showed fibrous tissue, microbleeds, consistent with the callus, and adjacent levels of sclerosis [5, 10]. This pathological result is similar to bone non-union, and this lesion is named “pseudarthrosis” [11]. Therefore, current literature reports generally believe that there is no bacterial infection in the bone tissue of ALs. In our case conventional bacterial culture did not detect any bacteria. Microscopic histopathological examination revealed fibrocartilage and bone tissue, fibrous necrosis, granulation tissue formation, and chronic inflammatory cell infiltration. However, through mNGS, Ewingella americana was identified in specimens collected from bone biopsies. Ewingela was first described by Grimont et al. in 1983 as a facultative anaerobic Gram-negative bacterium in the family Enterobacteriaceae [12]. The literature reports that humans have isolated this bacterium from blood, sputum, conjunctiva, and wounds [13]. Early studies have shown that Ewingela mainly found in immunocompromised individuals, such as postoperative patients, especially those undergoing cardiovascular surgery [14], patients with indwelling catheters [15], renal failure [16], and long-term hospitalization [13, 17]. Although the root cause of Ewingela AL was not described in previous cases, we conducted a comprehensive study of the patient’s medical history. The long course of the patient’s illness, irregular medication history, and long-term use of steroid hormones are likely the fundamental reasons for the AL of the Ewingela. According to reports, Ewingela is sensitive to ceftazidime, ampicillin/sulbactam (Unasyn), gentamicin, and cefepime, but resistant to ampicillin and ceftriaxone [13]. The patient’s condition significantly improved and lower back pain was significantly relieved by day 7 of hospital course with targeted use of cefoperazone after surgery. Fortunately, the patient was discharged from the hospital smoothly on the 16th day. The patient had no complications throughout the entire follow-up process. Of course, there is still controversy over whether Ewingela is a true pathogen or exists primarily as an opportunistic infection in the lesions of Als. However, mNGS has the characteristics of rapid and high sensitivity, providing us with a new concept for exploring the etiology of AL, which is of great significance for the identification of AL pathogens. Therefore, we suggest conducting mNGS on the diseased bone tissue of AL patients.

## Conclusion

We detected Ewingella americana in the bone biopsies of Andersson lesion (AL) in ankylosing spondylitis patient by mNGS, which plays an important role in further clarifying the etiology of AL. The mNGS is an important tool for further elucidating the etiology of AL.

## Data Availability

The datasets used and analysed during the current study are available from the corresponding author on reasonable request.

## Abbreviations

AL: Andersen’s lesion
AS: Ankylosing spondylitis
mNGS: Metagenomic Next-Generation Sequencing
NSAIDs: Nonsteroidal anti-inflammatory drugs
CRP: C-reactive protein
ESR: Erythrocyte sedimentation rate
WBC: White blood cell

## References

[1] Kabasakal Y, Garrett SL, Calin A. The epidemiology of spondylodiscitis in ankylosing spondylitis–a controlled study. Br J Rheumatol. 1996;35(7):660–3. 8670600 10.1093/rheumatology/35.7.660

[2] Kim DH, Kim SW, Lee SM. Complete Fusion of three lumbar vertebral bodies in Ankylosing Spondylitis. Korean J Neurotrauma. 2020;16(1):105–9. 32395459 10.13004/kjnt.2020.16.e1 PMC7192799

[3] Wang HF, Bi C, Chen ZQ. [Research progress on Andersson lesion in ankylosing spondylitis]. Zhonghua Wai Ke Za Zhi [Chinese J Surgery]. 2017;55(10):798–800. 29050182 10.3760/cma.j.issn.0529-5815.2017.10.020

[4] Bron JL, de Vries MK, Snieders MN, van der Horst-Bruinsma IE, van Royen BJ. Discovertebral (Andersson) lesions of the spine in ankylosing spondylitis revisited. Clin Rheumatol. 2009;28(8):883–92. 19294478 10.1007/s10067-009-1151-x PMC2711912

[5] Nikolaisen C, Nossent H. Early histology in ankylosing spondylitis related spondylodiscitis supports its inflammatory origin. Scand J Rheumatol. 2005;34(5):396–8. 16234189 10.1080/03009740510026625

[6] Dave BR, Ram H, Krishnan A. Andersson lesion: are we misdiagnosing it? A retrospective study of clinico-radiological features and outcome of short segment fixation. Eur Spine Journal: Official Publication Eur Spine Soc Eur Spinal Deformity Soc Eur Sect Cerv Spine Res Soc. 2011;20(9):1503–9. 10.1007/s00586-011-1836-0 PMC3175887 21559769

[7] Park YS, Kim JH, Ryu JA, Kim TH. The Andersson lesion in ankylosing spondylitis: distinguishing between the inflammatory and traumatic subtypes. J bone Joint Surg Br Volume. 2011;93(7):961–6. 10.1302/0301-620X.93B7.26337 21705571

[8] Shaik I, Bhojraj SY, Prasad G, Nagad PB, Patel PM, Kashikar AD, Kumar N. Management of Andersson Lesion in Ankylosing Spondylitis using the posterior-only Approach: a Case Series of 18 patients. Asian Spine J. 2018;12(6):1017–27. 30322255 10.31616/asj.2018.12.6.1017 PMC6284118

[9] Unsal E, Arici AM, Kavukçu S, Pirnar T. Andersson lesion: spondylitis erosiva in adolescents. Two cases and review of the literature. Pediatr Radiol. 2002;32(3):183–7. 12164351 10.1007/s00247-001-0629-8

[10] Poynter JA, Manukyan MC, Wang Y, Brewster BD, Herrmann JL, Weil BR, Abarbanell AM, Meldrum DR. Systemic pretreatment with dimethyloxalylglycine increases myocardial HIF-1α and VEGF production and improves functional recovery after acute ischemia/reperfusion. Surgery. 2011;150(2):278–83. 21801965 10.1016/j.surg.2011.06.006

[11] Calin A, Robertson D. Spondylodiscitis and pseudarthrosis in a patient with enteropathic spondyloarthropathy. Ann Rheum Dis. 1991;50(2):117–9. 1998387 10.1136/ard.50.2.117 PMC1004351

[12] Grimont PA, Farmer JJ 3rd, Grimont F, Asbury MA, Brenner DJ, Deval C. Ewingella americana gen.nov., sp.nov., a new Enterobacteriaceae isolated from clinical specimens. Ann De Microbiologie. 1983;134a(1):39–52. 10.1016/0769-2609(83)90102-3 6847036

[13] Khurana S, Chemmachel C, Saxena R. Ewingella americana Peritonitis in a patient on peritoneal Dialysis: a Case Report and Review of the literature. Case Rep Nephrol dialysis. 2020;10(3):147–53. 10.1159/000510147 PMC7747055 33363216

[14] Pien FD, Bruce AE. Nosocomial Ewingella americana bacteremia in an intensive care unit. Arch Intern Med. 1986;146(1):111–2. 3942442 10.1001/archinte.1986.00360130133018

[15] Maertens J, Delforge M, Vandenberghe P, Boogaerts M, Verhaegen J. Catheter-related bacteremia due to Ewingella americana. Clin Microbiol Infection: Official Publication Eur Soc Clin Microbiol Infect Dis. 2001;7(2):103–4. 10.1046/j.1469-0691.2001.00195.x 11298155

[16] Ryoo NH, Ha JS, Jeon DS, Kim JR, Kim HC. A case of pneumonia caused by Ewingella americana in a patient with chronic renal failure. J Korean Med Sci. 2005;20(1):143–5. 15716620 10.3346/jkms.2005.20.1.143 PMC2808562

[17] Devreese K, Claeys G, Verschraegen G. Septicemia with Ewingella americana. J Clin Microbiol. 1992;30(10):2746–7. 1400980 10.1128/jcm.30.10.2746-2747.1992 PMC270514

